# Discordant real‐world glycaemic outcomes with Omnipod™ 5 and MiniMed™ 780G in adults with type 1 diabetes: Why validated measures matter

**DOI:** 10.1111/dom.70480

**Published:** 2026-01-26

**Authors:** Panagiotis Pavlou, Ananthi Anandhakrishnan, Kleoniki I. Athanasiadou, Anna Brackenridge, Yuk‐Fun Liu, Dulmini Kariyawasam, Thomas Johnston, Rosarie Atkinson, Rebecca Hyslop, Siobhan Pender, Janaka Karalliedde, Sufyan Hussain

**Affiliations:** ^1^ Department of Diabetes and Endocrinology Guy's and St Thomas' NHS Foundation Trust London UK; ^2^ School of Cardiovascular and Metabolic Medicine and Science King's College London London UK; ^3^ Institute of Diabetes, Endocrinology and Obesity, King's Health Partners London UK

**Keywords:** CGM, HCL, hybrid closed loop system, real‐world evidence, type 1 diabetes

## 1 BACKGROUND

Hybrid closed loop (HCL) systems are the most advanced form of insulin therapy currently available for people with type 1 diabetes (pwT1D). However, despite their increasing use in clinical practice, there is a paucity of real‐world studies comparing different HCL systems.

To help address this evidence gap, we conducted a real‐world observational study comparing Omnipod™ 5 (OP‐5) with Medtronic MiniMed™ 780G (MT780G) in pwT1D. Our primary outcome was change from baseline laboratory glycated haemoglobin (HbA1c) after a follow‐up period post initiation of HCL therapy. We used HbA1c as the primary outcome to maximise objectivity in our comparison, taking into consideration reported inconsistencies in accuracy between CGMs.^1^, ^2^, ^3^

## 2 METHODS

We conducted a single‐centre observational study of adult pwT1D initiated on HCL therapy from January 2024 to January 2025 attending a university hospital. Adult users of MT780G or OP‐5 with baseline HbA1c 58–75 mmol/mol and available CGM data at baseline (pre‐HCL) and follow‐up were included. Baseline was defined as the most recent HbA1c and CGM data prior to HCL initiation (up to 6 months prior); follow‐up was the most recent HbA1c and CGM data at least 3 months after starting HCL. Haemodialysis and pregnancy were excluded. Details on HCL initiation, HbA1c analyser are included in Supporting Information.^4^

Demographic and clinical data extracted included age, sex, duration of diabetes, Index of Multiple Deprivation (IMD), baseline treatment modality, weight and HbA1c. CGM‐derived metrics included time‐in‐range (3.9–10.0 mmol/L), time‐in‐tight‐range (TITR, 3.9–7.8 mmol/L), time‐below‐range (TBR, <3.9 mmol/L), time‐above‐range (TAR, >10), glucose management indicator (GMI), mean glucose and glycaemic variability (CV). Missing data were minimal (6.3%) and were handled by listwise deletion.

Baseline characteristics were compared between OP‐5 and MT780G users. Continuous variables were assessed for normality within each group using histograms, Q–Q plots, and Shapiro–Wilk tests. Where both groups were normally distributed, Welch's t‐test was used. For skewed variables, we used the Wilcoxon rank‐sum test.

Within each group, change from baseline was measured for HbA1c, TIR, TITR, CV, GMI and TBR. For normal variables, paired t‐test was used; otherwise, Wilcoxon signed‐rank test was applied.

The primary outcome was follow‐up HbA1c post HCL. We used analysis of covariance (ANCOVA) with HbA1c as the dependent variable and pump type as the main exposure, adjusting for baseline HbA1c, diabetes duration, age and IMD. Details on secondary endpoint analysis are included in Supporting Information. We assessed homogeneity of variance using Levene's test to model residuals across groups. In all models, Levene's test was non‐significant.

TITR exhibited a right‐skewed distribution and did not meet normality assumptions required for ANCOVA. Therefore, TITR was log‐transformed using the natural logarithm to approximate a normal distribution and allow parametric modelling. Model coefficients were estimated on the log scale and subsequently back‐transformed to aid clinical interpretation by exponentiating the regression coefficient (e^β) to obtain the ratio of geometric means of TITR between groups. This ratio was then applied to the observed mean TITR to express the effect as an approximate absolute difference in percentage points at follow‐up.

TBR data were highly skewed and zero‐inflated; therefore, we analysed between‐group differences using Wilcoxon rank‐sum test and within‐group changes using Wilcoxon signed‐rank test.

All analyses were performed in R (version 4.4.2). A two‐tailed *p* < 0.05 was considered statistically significant.

## 3 RESULTS

A total of 129 adults (67% female, 33% male) using HCL were included (48 on MT780G, 81 on OP‐5) with median follow up (range) 15^5^, ^6^, ^7^, ^8^, ^9^, ^10^, ^11^, ^12^, ^13^, ^14^, ^15^, ^16^, ^17^, ^18^, ^19^, ^20^, ^21^ months. Groups had similar IMD; however, the OP‐5 group was significantly younger (35 vs. 50 years, *p* < 0.001) with shorter diabetes duration (20.5 vs. 28 years, *p* < 0.001) and lower BMI (26.9 vs. 29.7 kg/m^2^, *p* = 0.003) at baseline (Table 1). No significant differences between groups were present in HbA1c, CV and GMI at baseline. Those initiated on OP‐5 had lower TITR (23.4 vs. 28.1%; *p* = 0.021), TIR (42.2 vs. 48.4%; *p* = 0.028) and weight (73.3 vs. 82.0 kg; *p* = 0.012) at baseline (Table 1). There was no difference in time spent in automated mode between systems (*p* = 0.59) (see Supporting Information). Regarding the type of CGM used, MT780G users were on Medtronic Guardian™ 4 (75%) and Simplera™ (25%), while those on OP‐5 used Dexcom™ G6 (87%), Dexcom™ G7 (5%) and Freestyle Libre™ 2 Plus (8%).

**TABLE 1:** Baseline population characteristics.

Characteristic	OP‐5 (*n* = 81)	MT780G (*n* = 48)	Overall (*N* = 129)	Difference (95% CI); *p*‐value
Number of participants	81	48	129	
Age (years), median (range)	35.0 (21.0–67.0)	50.0 (24.0–66.0)	41.0 (21.0–67.0)	−15.00 (−20.00 to −7.00); *p* < 0.001
Duration diabetes (years), median (range)	20.5 (1.0–51.0)	28.0 (6.0–54.0)	23.0 (1.0–54.0)	−7.50 (−15.00 to −4.00); p < 0.001
IMD, median (IQR)	5.0 (3.0–7.0)	5.5 (3.0–8.8)	5.0 (3.0–8.0)	−0.50 (−3.00 to 2.00); *p* = 0.507
Female, *n* (%)	54 (66.7%)	33 (68.8%)	87 (67.4%)	−0.02 (−0.19 to 0.15); *p* = 0.807
Ethnicity				
White, *n* (%)	50 (61.7%)	37 (77.1%)	87 (67.4%)	−0.15 (−0.31 to 0.01); *p* = 0.072
Black, *n* (%)	7 (8.6%)	2 (4.2%)	9 (7.0%)	0.04 (−0.04 to 0.13); *p* = 0.335
Asian, *n* (%)	5 (6.2%)	2 (4.2%)	7 (5.4%)	0.02 (−0.06 to 0.10); *p* = 0.627
Mixed, *n* (%)	2 (2.5%)	4 (8.3%)	6 (4.7%)	−0.06 (−0.14 to 0.03); *p* = 0.126
Other, *n* (%)	17 (21.0%)	3 (6.2%)	20 (15.5%)	0.15 (0.04 to 0.26); *p* = 0.0254
Baseline CSII, *n* (%)	43 (53.1%)	36 (75.0%)	79 (61.2%)	−0.22 (−0.38 to −0.06); *p* = 0.0135
Baseline MDI, *n* (%)	35 (43.2%)	12 (25.0%)	47 (36.4%)	0.18 (0.02 to 0.35); *p* = 0.0378
Structured education, *n* (%)	42 (51.9%)	20 (41.7%)	62 (48.1%)	0.10 (−0.08 to 0.28); *p* = 0.263
HbA1c baseline, mean ± SD	69.7 ± 9.4	68.1 ± 6.8	69.1 ± 8.5	−1.00 (−2.00 to 3.00); *p* = 0.61
CV baseline, mean ± SD	36.6 ± 6.9	37.5 ± 5.7	36.9 ± 6.4	−1.15 (−3.10 to 0.90); *p* = 0.248
TIR baseline, mean ± SD	42.2 ± 16.0	48.4 ± 15.1	44.5 ± 15.9	−6.26 (−11.80 to −0.69); p = 0.028
TITR baseline, mean ± SD	23.4 ± 11.6	28.1 ± 10.5	25.1 ± 11.4	−4.75 (−8.76 to −0.73); p = 0.021
Weight baseline, mean ± SD	73.3 ± 17.4	82.0 ± 18.3	76.5 ± 18.1	−8.68 (−15.40 to −1.93); *p* = 0.0123
BMI baseline, mean ± SD	26.9 ± 6.7	29.7 ± 6.2	27.9 ± 6.6	−2.75 (−5.80 to −0.35); *p* = 0.00371

*Note*: Data are presented as mean ± standard deviation (SD), median (range), median (interquartile range) or *n* (%), as appropriate. Differences are shown as OP5–780G.

Abbreviations: BMI, body mass index; CV, coefficient of variation; HbA1c, glycated haemoglobin; IMD, Index of Multiple Deprivation; MT780G, Medtronic MiniMed™ 780G; OP‐5, Omnipod™ 5; TIR, time in range (3.9–10.0 mmol/L); TITR, time in tight range (3.9–7.8 mmol/L).

Both HCL systems led to substantial improvements from baseline in most glycaemic outcomes (Supporting Information). HbA1c decreased by 9.7 mmol/mol (95% CI 12.0, 7.4; *p* < 0.001) in OP‐5 users and 8.8 mmol/mol (95% CI 11.3, 6.2; *p* < 0.001) in MT780G users. TIR increased by 17.6 percentage points (pp) (95% CI 14.2, 20.9; *p* < 0.001) in the OP‐5 group and 23.8 pp (95% CI 18.6, 29.0; *p* < 0.001) in the MT780G group. TITR increased by 13.6 pp (95% CI 11.2, 16.0; *p* < 0.001) with OP‐5 and 19.0 pp (95% CI 14.8, 23.3; *p* < 0.001) with MT780G. CV decreased significantly with MT780G (−6.2%, 95% CI −8.0, −4.4; *p* < 0.001) but not with OP‐5 (−0.7%, 95% CI −2.3, 0.9; *p* = 0.39). GMI improved in both groups (OP‐5: −6.4 mmol/mol, 95% CI −8.1, −4.8; 780G: −9.2 mmol/mol, 95% CI −11.5, −7.1; both *p* < 0.001). Median TBR decreased by 1 pp in both groups (all *p* ≤ 0.02).

**FIGURE 1:**
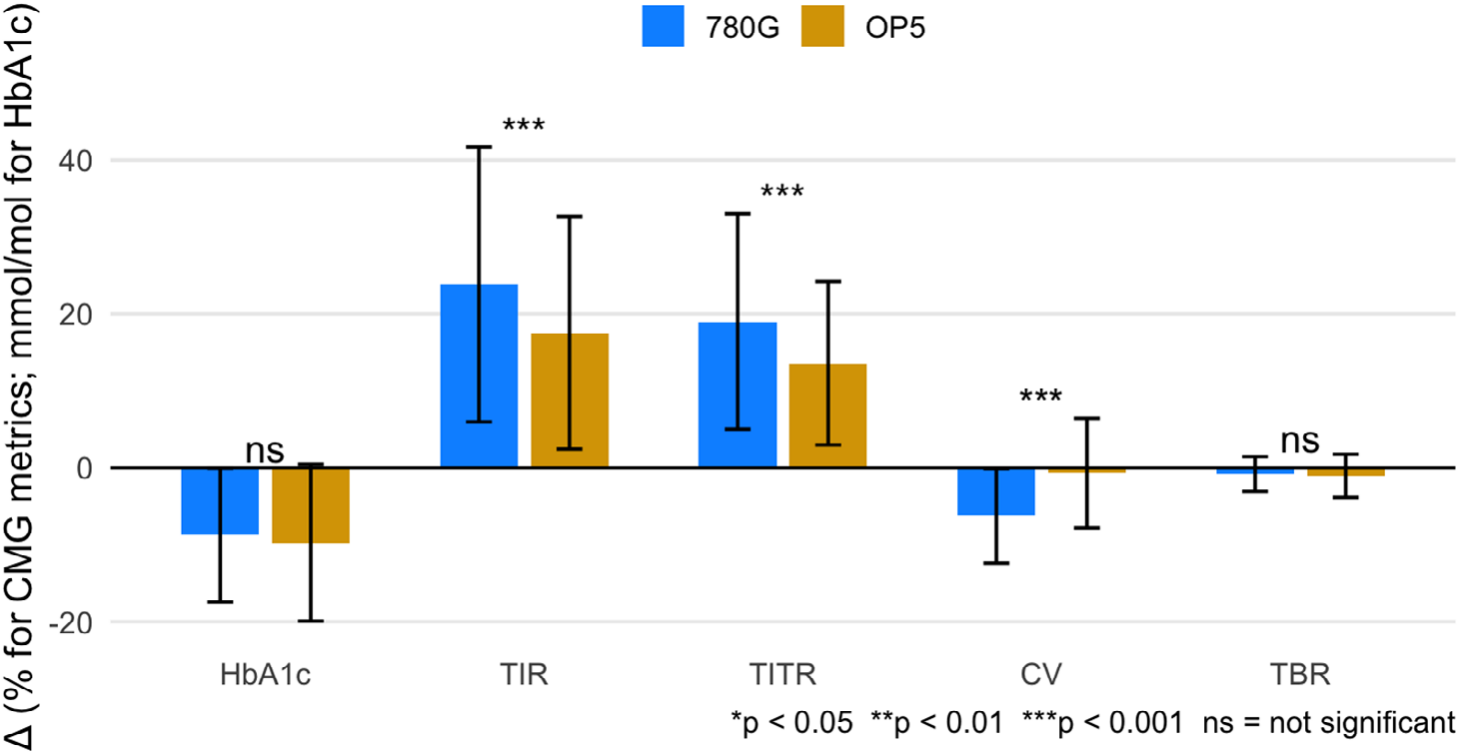
Changes from baseline in glycaemic outcomes by system (error bars = SD).

In the primary analysis, there was no significant difference in follow‐up HbA1c between systems after adjusting for baseline HbA1c, diabetes duration, age and IMD (adjusted mean difference MT780G–OP‐5: −0.51 mmol/mol; 95% CI −4.24, 3.22; *p* = 0.73) (Figure 1). Sensitivity analyses incorporating baseline treatment modality and BMI did not materially alter the direction, magnitude, or statistical significance of the primary findings (Table S3).

In secondary analysis, MT780G was associated with superior outcomes in certain CGM‐derived metrics (Figure 1). Follow‐up TIR was 11.0 pp. higher with MT780G than OP‐5 (95% CI 6.4, 15.6; *p* < 0.001), adjusted for baseline HbA1c, diabetes duration, age and IMD. For post‐HCL log‐transformed TITR, the adjusted difference (OP‐5–MT780G) was −0.18 (95% CI 0.06, 0.31; *p* = 0.0015), corresponding to a TITR of approximately 4.9 pp. higher for MT780G; this calculated by exponentiating the regression coefficient to obtain the ratio of geometric means of TITR between groups which was subsequently anchored to the reference group mean. Follow‐up CV was significantly lower with MT780G (−4.9%, 95% CI −6.6, −3.2; *p* < 0.001). Post‐HCL GMI was lower with MT780G (−4.5 mmol/mol, 95% CI −2.8, −6.2; *p* < 0.001). Between‐group comparison of TBR showed no significant difference (*p* = 0.79).

## 4 CONCLUSION

Overall, both systems improved glycaemic levels substantially compared to baseline, in line with previous real‐world findings.^5^, ^6^ There was no statistically significant difference in HbA1c at follow‐up between OP‐5 and MT780G after adjusting for baseline HbA1c, duration of diabetes, age and IMD. MT780G was associated with significantly higher TIR and TITR and lower CV than OP‐5.

We acknowledge that, as this was a non‐randomised observational study, causal inference cannot be established. Another limitation is that several factors that may play a role in glycaemic outcomes, such as user behaviour, patient‐reported outcome measures, baseline CGM types and HCL system settings including glucose targets, autocorrection behaviour, and insulin delivery parameters, were not available. Furthermore, the two groups had unequal sizes and substantial baseline differences in age, diabetes duration, TIR, TITR, BMI and treatment modality. Although ANCOVA adjustment was applied, these imbalances may still influence comparative outcomes and residual confounding cannot be excluded. It is also worth noting that in large real‐world datasets, it was observed that older MT780G users can achieve higher TIR compared with younger users, making age imbalance a plausible contributor to observed differences in TIR.^7^ An additional limitation is that the distribution of CGM types was not uniform across groups, and this presents an inherent challenge in interpreting between‐system CGM metrics. Finally, we acknowledge that our study was not designed as an equivalence or non‐inferiority study and as such, no equivalence margin is specified; therefore, the study may be underpowered to detect small but clinically relevant differences.

Recent meta‐analyses investigating HCL systems also identified differences in glycaemic outcomes between systems based on comparisons from pooled data of CGM‐derived metrics, a finding also echoed by a recent review of real‐world outcomes.^8^, ^9^, ^10^, ^11^ Outcomes with HCL systems are baseline dependent. Higher HbA1c is associated with bigger reductions, and baseline glycaemia influences time‐in‐range attainment.^11^, ^12^, ^13^, ^14^ Pooled and real‐world analyses do not account for these baseline differences. Furthermore, CGM metrics are not validated across different sensors, while data indicating significant discrepancies in accuracy have emerged.^1^, ^2^, ^3^

Two recent comparative studies demonstrated significant intersystem discordance in CGM accuracy. Specifically, it was observed that Medtronic Simplera™ overestimated TIR in comparison to Dexcom™ G7 and Freestyle Libre™ 3 and had lower accuracy in normoglycaemia and hyperglycaemia.^1^, ^2^ Given these observations, the validity of using CGM metrics as surrogate markers to compare glycaemic outcomes across HCL systems warrants renewed scrutiny. While we acknowledge that all commercially available CGMs have U.S. Food and Drug Administration (FDA) and CE (Conformité Européenne) mark approval, we do not intend to challenge regulatory approval of CGM systems, but to highlight that clinically meaningful inter‐system differences in CGM‐derived metrics have been demonstrated even among approved devices, as documented in recent comparator studies and International Federation of Clinical Chemistry and Laboratory Medicine (IFCC) discussions.^15^

HbA1c is the most extensively validated biomarker of glycaemic management and complication risk.^16^, ^17^ Laboratory assays conform to fixed international reference methods, maintain low inter‐lab variation, low analytical coefficient of variation, and are comparable between countries, instruments, and reagents.^18^, ^19^

Despite similar baseline HbA1c, both systems achieved similar HbA1c reductions, even though MT780G delivered greater improvements in TIR, TITR and CV. This divergence between laboratory HbA1c and CGM‐derived metrics suggests that the apparent between‐system differences may reflect discordance in CGM accuracy rather than true biological effect on glycaemia. It is important to note, however, that the observed discordance could reflect both CGM characteristics and genuine differences in glycaemic profiles, which are components we could not disentangle in our study. Our findings are constrained by the real‐world observational design, imbalance in baseline CGM metrics and absence of detailed data on HCL settings. Due to these limitations, no causal relationship can be established and our findings should be interpreted as hypothesis‐generating. Future real‐world comparisons should prioritise validated outcomes such as laboratory HbA1c, supported by consistent blinded CGM across study arms, and be underpinned by harmonised CGM accuracy standards to ensure robust and interpretable comparisons between CGM systems.^3^, ^20^, ^21^

## AUTHOR CONTRIBUTIONS

Sufyan Hussain, Janaka Karalliedde, Ananthi Anandhakrishnan and Panagiotis Pavlou conceived the concept for this manuscript. Sufyan Hussain and Janaka Karalliedde supervised the project. Panagiotis Pavlou, Sufyan Hussain and Janaka Karalliedde wrote the manuscript, and Panagiotis Pavlou and Sufyan Hussain performed the literature investigation. Panagiotis Pavlou and Ananthi Anandhakrishnan collected the data. Panagiotis Pavlou performed the data analysis and prepared the figures and tables. All authors critically reviewed, edited and approved the final manuscript.

## CONFLICT OF INTEREST STATEMENT

Sufyan Hussain has served on the advisory board for Tandem, Dexcom, Medtronic; undertaken non‐promotional educational and/or consultancy work for Abbott UK, Insulet, Dexcom, Medtronic and Roche. All other authors report no conflict of interest.

## Supporting information


**Data S1.** Supporting Information.

## Data Availability

The data that support the findings of this study are available from the corresponding author upon reasonable request.
