# Supplementary material for: Discordant real‐world glycaemic outcomes with Omnipod™ 5 and MiniMed™ 780G in adults with type 1 diabetes: Why validated measures matter
Source: Diabetes Obes Metab. 2026 Jan 26;28(4):3424–8. doi: 10.1111/dom.70480 (PMC12992204; doi:10.1111/dom.70480)

**HCL initiation:**

HCL initiation was based on clinical indications consistent with current best practice guidelines [8]. Choice of HCL system was dependent upon patient preference and system characteristics such total insulin capacity. Sensors used with MT780G were Guardian 4^TM^ and Simplera^TM^. Those on OP-5 used Dexcom G6^TM^, Dexcom G7^TM^ and Freestyle Libre 2 Plus^TM^.

**HbA1c analyzer:**The type of HbA1c analyzer used by our clinic is Hb9210 Premier HbA1c analyzer, Menarini Diagnostics.

**Secondary analysis methodology:**

For the secondary exploratory analysis, ANCOVA models were used to examine follow-up TIR, log-transformed TITR, CV, GMI and weight, each adjusted for its respective baseline value; the TIR and TITR models additionally adjusted for baseline HbA1c, diabetes duration, age and IMD. TITR was log-transformed to achieve normality and allow multiplicative interpretation of effects.

**Table S1. Within-group changes from baseline to follow-up**

| **Variable** | **Group** | **n** | **Change (follow-up – baseline)** | **95% CI** | **p-value** |
| --- | --- | --- | --- | --- | --- |
| HbA1c (mmol/mol) | OP5 | 80 | −9.71 | −12.0 to −7.44 | <0.001 |
|  | 780G | 48 | −8.75 | −11.3 to −6.23 | <0.001 |
| TIR (%) | OP5 | 81 | +17.6 | +14.2 to +20.9 | <0.001 |
|  | 780G | 48 | +23.8 | +18.6 to +29.0 | <0.001 |
| TITR (%) | OP5 | 79 | +13.6 | +11.2 to +16.0 | <0.001 |
|  | 780G | 44 | +19.0 | +14.8 to +23.3 | <0.001 |
| CV (%) | OP5 | 81 | −0.68 | −2.26 to +0.90 | 0.39 |
|  | 780G | 47 | −6.24 | −8.04 to −4.44 | <0.001 |
| GMI (mmol/mol) | OP5 | 80 | −6.38 | −8.10 to −4.75 | <0.001 |
|  | 780G | 47 | −9.15 | −11.5 to −7.05 | <0.001 |
| TBR (%) | OP5 | 81 | −1.00 | −2.50 to −0.50 | 0.0038 |
|  | 780G | 48 | −1.00 | −2.00 to 0 | 0.0155 |

*Positive values indicate an increase from baseline; negative values indicate a decrease*

**Table S2. Adjusted between-group differences (MT780G vs OP-5)**

| **Outcome** | **Model adjustment** | **Difference (780G – OP5)** | **95% CI** | **p-value** | **Interpretation** |
| --- | --- | --- | --- | --- | --- |
| HbA1c (mmol/mol) | Baseline HbA1c, duration, age, IMD | −0.51 | −4.24 to 3.22 | 0.73 | No difference in HbA1c |
| TIR (%) | Baseline TIR, baseline HbA1c, duration, age, IMD | +11.0 | +6.36 to +15.6 | <0.001 | Higher TIR with 780G |
| log(TITR)† | log(TITR baseline) | +0.18 | +0.06 to +0.31 | 0.0015 | Approximately 4.9% higher TITR with MT780G |
| CV (%) | Baseline CV | −4.9 | −6.63 to −3.16 | <0.001 | Lower glycaemic variability with MT780G |
| GMI (mmol/mol) | Baseline GMI | −4.50 | −6.25 to −2.76 | <0.001 | Lower GMI with MT780G |
| Weight (kg) | Baseline weight | +1.97 | −1.36 to +5.29 | 0.25 | No significant difference in weight |
| TBR* (%)  (median) | Unadjusted | 0 | 0 to 1 | 0.79 | Similar hypoglycaemia burden |
| Time in automated mode*¶ (%)(median) | Unadjusted | 1 | (-2.0 to 0.99) | 0.59 | No significant difference in time in automated mode |
| Time sensor active*¶ (%)(median) | Unadjusted | 1 | (-1.9, 0.5) | 0.33 | No significant difference in time in sensor active |

**Wilcoxon rank-sum test was used for TBR, Time in automated mode and time sensor active comparisons. All other comparisons were made using ANCOVA.*

*¶Time in automated mode is a proportion of the time the sensor is active*

†*TITR was log-transformed using the natural logarithm to approximate a normal distribution and allow parametric modelling. The absolute difference in original scale corresponds to a TITR of approximately 4.9% higher for MT780G by exponentiating the regression coefficient and anchoring to the reference group mean.*

*Abbreviations: MT780=Medtronic 780G^TM^, OP-5=Omnipod 5^TM^, HbA1c=Haemoglobin A1c, TIR=Time in range, TITR=Time in tight range, CV=Glucose variability, GMI=Glucose management indicator, TBR= Time below range*

**Table S3**. Sensitivity analysis: adjusted between-group differences (MT780G vs OP-5)

| Outcome | Model adjustment | Difference (780G – OP-5) | 95% CI | p-value | Interpretation |
| --- | --- | --- | --- | --- | --- |
| HbA1c (mmol/mol) | Baseline HbA1c, duration, age, IMD, baseline treatment | -0.99 | -4.75 to 2.77 | 0.64 | No difference in HbA1c |
| HbA1c (mmol/mol) | Baseline HbA1c, duration, age, IMD, BMI | -1.04 | -5.04 to 2.97 | 0.69 | No difference in HbA1c |
| HbA1c (mmol/mol) | Baseline HbA1c, duration, age, IMD, baseline treatment, BMI | -1.37 | -5.39 to 2.65 | 0.60 | No difference in HbA1c |
| TIR (%) | Baseline TIR, duration, age, IMD, BMI | +11.5 | +6.52 to +16.5 | <0.001 | Higher TIR with 780G |
| TIR (%) | Baseline TIR, duration, age, IMD, baseline treatment | +11.4 | +6.76 to +16.1 | <0.001 | Higher TIR with 780G |
| TIR (%) | Baseline TIR, duration, age, IMD, baseline treatment, BMI | +11.8 | +6.82 to +16.9 | <0.001 | Higher TIR with 780G |

**Notes:***All analyses were performed using ANCOVA*

*Abbreviations: MT780G, Medtronic MiniMed™ 780G; OP-5, Omnipod™ 5; HbA1c, glycated haemoglobin; TIR, time in range; IMD, Index of Multiple Deprivation.*

**Supplementary figures:**


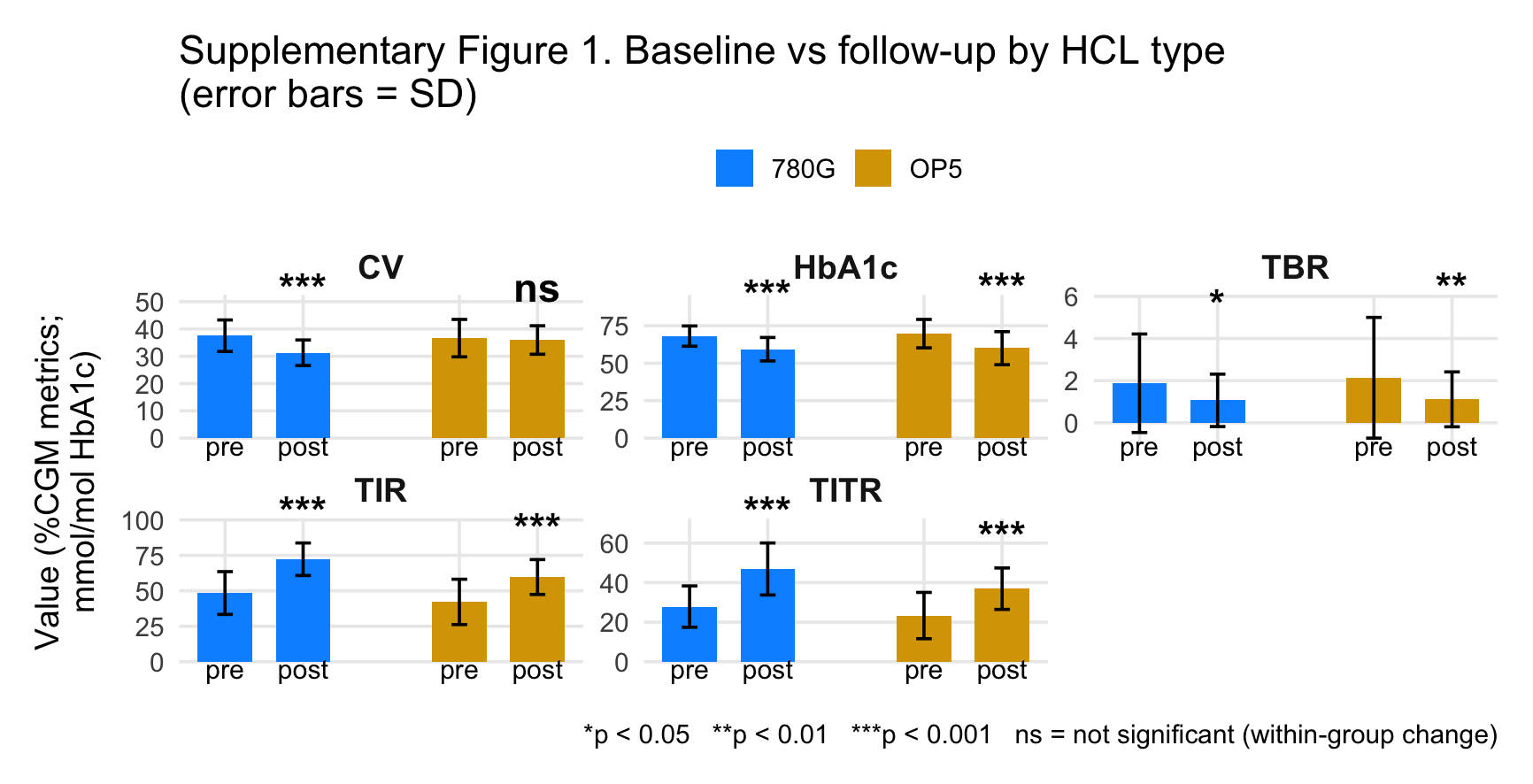

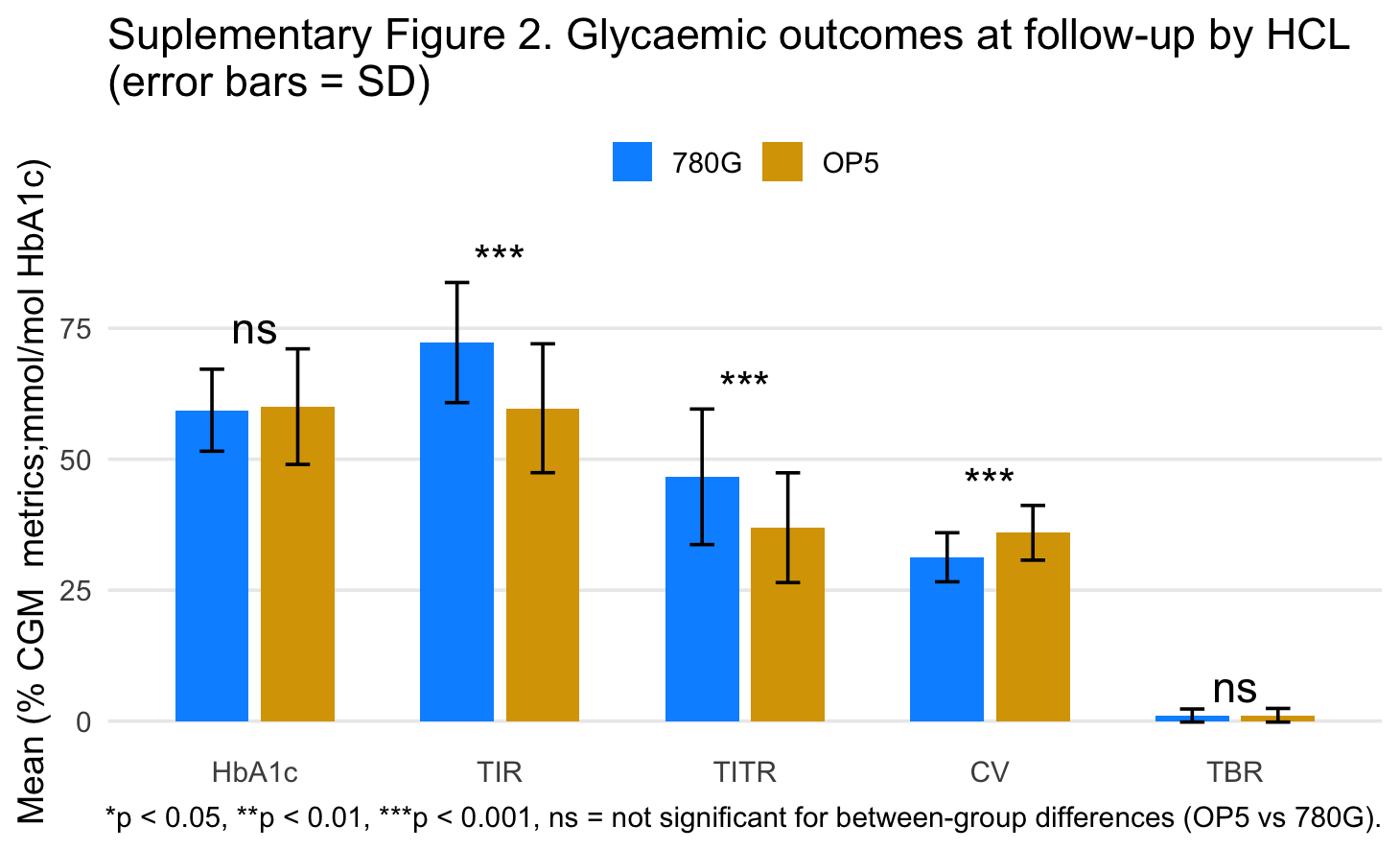

Supplement: Supplementary file 1 — Data S1. Supporting Information. [file DOM-28-3424-s001.docx]
